# Genomic analysis in Chilean patients with suspected Rett syndrome: keep a broad differential diagnosis

**DOI:** 10.3389/fgene.2024.1278198

**Published:** 2024-03-18

**Authors:** Florencia Brito, Catalina Lagos, Jessica Cubillos, Joan Orellana, Mallen Gajardo, Daniela Böhme, Gonzalo Encina, Gabriela M. Repetto

**Affiliations:** ^1^ Rare Diseases Program, Center for Genetics and Genomics, Institute of Sciences and Innovation in Medicine, Facultad de Medicina, Clínica Alemana Universidad del Desarrollo, Santiago, Chile; ^2^ Fundación por Ellas y Ellos Síndrome de Rett, Santiago, Chile; ^3^ Escuela de Ingeniería, Facultad de Ciencias Físicas y Matemáticas, Universidad de Chile, Santiago, Chile; ^4^ Biosoluciones-UDD, Santiago, Chile

**Keywords:** Rett syndrome, MeCP2, CpG methyl-binding protein, neurodevelopmental disorders, gene panel, genomic testing

## Abstract

**Introduction:** Rett syndrome (RTT, MIM #312750) is a rare genetic disorder that leads to developmental regression and severe disability and is caused by pathogenic variants in the *MECP2* gene. The diagnosis of RTT is based on clinical features and, depending on resources and access, on molecular confirmation. There is scarce information on molecular diagnosis from patients in Latin America, mostly due to limited availability and coverage of genomic testing. This pilot study aimed to implement genomic testing and characterize clinical and molecular findings in a group of Chilean patients with a clinical diagnosis of RTT.

**Methods:** Twenty-eight patients with suspected RTT underwent characterization of phenotypic manifestations and molecular testing using Clinical Exome Solution^TM^ CES_V2 by SOPHiA Genetics. Data was analyzed using the commercial bioinformatics platform, SOPHiA DDM^TM^. A virtual panel of 34 genes, including *MECP2* and other genes that are in the differential diagnosis of RTT, was used to prioritize initial analyses, followed by evaluation of the complete exome sequence data.

**Results:** Twelve patients (42.8% of participants) had variants in *MECP2*, of which 11 (39.2%) were interpreted as pathogenic/likely pathogenic (P/LP), thus confirming the diagnosis of RTT in them. Eight additional patients (28.5%) harbored ten variants in nine other genes. Four of these variants were interpreted as P/LP (14.2%) (*GRIN2B, MADD, TRPM3* and *ZEB2*) resulting in alternative neurodevelopmental diagnoses, and six were considered of uncertain significance. No evident candidate variant was found for eight patients.

**Discussion:** This study allowed to reach a diagnosis in half of the participants. The diagnosis of RTT was confirmed in over a third of them, while others were found to have alternative neurodevelopmental disorders. Further evaluation is needed to identify the cause in those with negative or uncertain results. This information is useful for the patients, families, and clinicians to guide clinical management, even more so since the development of novel therapies for RTT. We also show the feasibility of implementing a step-wide approach to genomic testing in a setting with limited resources.

## Introduction

Rett syndrome (RTT, MIM #312750) is a rare neurodevelopmental genetic disorder that leads to severe disability. It affects almost every aspect of a patient’s life: their ability to talk, walk, eat, and even breathe normally (Kaur and Christodoulou, 1993; Neul et al., 2010). First described in 1966 by Dr. Andreas Rett (Rett, 1966), the frequency of RTT is estimated to be 1:10,000 live female births (Anderson et al., 2014).

The diagnosis of RTT is based on clinical features, with the hallmark of neurodevelopmental regression in infancy with subsequent stabilization or potential improvement, associated with main and supportive criteria, and the absence of exclusion criteria (Neul et al., 2010). Accordingly, two main phenotypes are described in females: “Typical” or “Classical” RTT, with developmental regression, usually between 1 and 4 years of age, and the presence of the four main criteria: loss of purposeful manual skills and acquired language, gait abnormalities, and development of manual stereotypies (such as hand washing, clapping, wringing or others) (Kaur and Christodoulou, 1993; Hagberg, 2002; Neul et al., 2008; Neul et al., 2010). However, if only some main and supportive criteria are met, patients are diagnosed as “Atypical” or “Variant” RTT, which can be either milder or more severe than typical RTT (Neul et al., 2010). Patients with atypical Rett syndrome can have diverse phenotypic manifestations, including conserved speech, early seizure, and congenital forms (Neul et al., 2008; Neul et al., 2010). Both typical and atypical RTT can present comorbidities, such as epilepsy, altered sleep pattern, bruxism and scoliosis (Lee et al., 2001; Hagberg, 2002; Neul et al., 2008). In males, there is broader phenotypic variability, ranging from miscarriage, stillbirth, severe early postnatal encephalopathy to survival with intellectual disability (Kaur and Christodoulou, 1993; Kankirawatana et al., 2006; Neul et al., 2010).

RTT is caused by pathogenic variants in the *MECP2* gene, located on the long arm of the X chromosome (Xq28) (Kaur and Christodoulou, 1993; Neul et al., 2010). *MECP2* encodes the Methyl-CpG binding protein 2, involved in neuronal growth, maturation and dendritic branching. *MECP2* variants are identified in up to 80%–90% of patients with the typical forms of RTT and in 40%–60% of those with the atypical form, and most occur as *de novo* events (Neul et al., 2008; Neul et al., 2010). Variants in other genes can cause related phenotypes, such as *CDKL*5 (cyclin-dependent-kinase-like 5; MIM #300203), which is altered in 3%–10% of atypical forms (Scala et al., 2005; Archer et al., 2006; Sartori et al., 2009; Guerrini and Parrini, 2012) and *FOXG1* (Forkhead box G1; MIM #164874), especially in congenital forms (Ariani et al., 2008; Mencarelli et al., 2010; Philippe et al., 2010; Guerrini and Parrini, 2012). Furthermore, molecular discoveries and the increased use of clinical genomic testing have led to the identification of neurodevelopmental disorders that may present clinical findings overlapping RTT. *STXBP1, TCF4, SCN2A, WDR45* and *MEF2C* are among the most common genes identified in patients with RTT-like phenotypes (Olson et al., 2015; Lopes et al., 2016; Lucariello et al., 2016; Vidal et al., 2017; Vidal et al., 2019a; Vidal et al., 2019b; Cogliati et al., 2019; Iwama et al., 2019; Schönewolf-Greulich et al., 2019).

Clinical manifestations of RTT are variable between patients, and some may be underdiagnosed when expecting complete fulfillment of the diagnostic criteria. Phenotypic overlap with other conditions may also pose diagnostic challenges. These situations can cause diagnostic and therapeutic errors, and lead to confusion in patients and clinicians. Molecular studies can thus help clarify the diagnosis of RTT. In addition, ongoing therapeutic developments make accurate diagnostic confirmation even more crucial for patients with RTT (Palmieri et al., 2023).

Scarce information exists on molecular diagnosis of RTT in patients from Latin America, mostly due to limited availability and coverage of genomic testing (Taruscio et al., 2023). Motivated by a request from the leadership of the local RTT patient organization in Chile and in collaboration with them, we developed this pilot study. We aimed to characterize the clinical and molecular findings of a group of patients with clinical diagnosis of RTT in Chile, and to assess the feasibility of implementing molecular testing in a country in which next-generation sequencing (NGS) is unavailable in the healthcare system and accessible only to patients who can afford to make out-of-pocket payments for testing performed abroad (Encina et al., 2019).

## Materials and methods

Chilean patients from the RTT Foundation “Caminamos por Ellas y Ellos-Síndrome de Rett Chile” with clinical diagnosis of RTT made by their physicians but without molecular confirmation, were invited to participate in this study through the Foundation. The study was approved by the Ethics Research Committee at Facultad de Medicina, Clinica Alemana Universidad del Desarrollo in Santiago, Chile, and parents or guardians gave written informed consent.

### Clinical information

To characterize the phenotypic manifestations of the participants, clinical information was collected through a standardized questionnaire based on RTT clinical criteria (Neul et al., 2010) and completed by a parent or guardian.

### Molecular analysis

To identify causal genetic variants, patients underwent clinical exome sequencing (CES). Genomic DNA was extracted from blood using DNeasy Blood and Tissue kit (QIAGEN, Germany). DNA quantification and integrity were analyzed using Qubit dsDNA BR (Life Technologies, CA, United States) and TapeStation 2200 gDNA (Agilent, CA, United States), respectively. Genomic DNA libraries and CES were performed using the Clinical Exome Solution™ v2 kit (SOPHiA Genetics, Lausanne, Switzerland), which captures exons from 4,490 genes known to cause monogenic diseases. Samples were sequenced on a MiSeq instrument (Illumina, CA, United States) at Universidad del Desarrollo, according to the manufacturer’s recommendations.

Data were analyzed with the SOPHiA DDM™ platform (SOPHiA Genetics) using hg19 (GRCh37) as reference. Variants were called, annotated and classified in this platform using its proprietary algorithm based on quality, frequency and functional impact. Information on the patients’ clinical phenotype was captured using Human Phenotype Ontology (HPO) terms (Köhler et al., 2021) (https://hpo.jax.org ). A virtual panel of 34 genes, including *MECP2* and other genes that are in the differential diagnosis of RTT, was created to prioritize analysis (Table 1). This list of genes was generated considering the ClinGen Rett and Angelman-like Disorders Variant Curation Expert Panel recommendations (Rehm et al., 2015) (https://www.clinicalgenome.org/affiliation/50022), data from the literature (Lopes et al., 2016; Lucariello et al., 2016; Vidal et al., 2017; Vidal et al., 2019b; Cogliati et al., 2019; Iwama et al., 2019; Schönewolf-Greulich et al., 2019), and overlapping genes included in panels for RTT and related conditions offered by clinical laboratories listed in the Genetic Testing Registry (https://www.ncbi.nlm.nih.gov/gtr/all/tests/?term=Rett). For mutation negative cases, the analysis was expanded to the 4,490 genes in the CES panel, in search of other potential causes of neurodevelopmental genetic disorders.

**TABLE 1:** Genes associated with Rett syndrome or similar neurodevelopmental conditions prioritized for analysis in this study.

Gene	Chr region	OMIM Gene ID	OMIM Phenotype ID	OMIM Phenotype
*ADSL*	22q13	608222	103050	Adenylosuccinase deficiency
*ALDH5A1*	6p22.3	610045	271980	Succinic semialdehyde dehydrogenase deficiency
*ARX*	Xp21.3	300382	308350	Developmental and epileptic encephalopathy 1
			300419	Intellectual developmental disorder, X-linked 29
*ATRX*	Xq21.1	300032	309580	Intellectual disability-hypotonic facies syndrome, X-linked
*CDKL5*	Xp22.13	300203	300672	Developmental and epileptic encephalopathy 2
*CNTNAP2*	7q35-q36.1	604569	610042	Pitt-Hopkins like syndrome 1
*CTNNB1*	3p22.1	116806	615075	Neurodevelopmental disorder with spastic diplegia and visual defects
***DDX3X***	**Xp11.4**	**300160**	**300958**	**Intellectual developmental disorder, X-linked syndromic, Snijders Blok type**
*DYRK1A*	21q22.13	600855	614104	Intellectual developmental disorder, autosomal dominant 7
*EHMT1*	9q34.3	607001	610253	Kleefstra syndrome 1
*FOLR1*	11q13.4	136430	613068	Neurodegeneration due to cerebral folate transport deficiency
*FOXG1*	14q12	164874	613454	Rett syndrome, congenital variant
*GRIA3*	Xq25	305915	300699	Intellectual developmental disorder, X-linked syndromic, Wu type
*GRIN2A*	16p13.2	138253	245570	Epilepsy, focal, with speech disorder and with or without impaired intellectual development
			616139	Developmental and epileptic encephalopathy 27
***GRIN2B***	**12p13.1**	**138252**	**613970**	**Intellectual developmental disorder, autosomal dominant 6, with or without seizures**
HDAC8	Xq13.1	300269	300882	Cornelia de Lange syndrome 5
IQSEC2	Xp11.22	300522	309530	Intellectual developmental disorder, X-linked 1
*MBD5*	2q23.1	611472	156200	Intellectual developmental disorder, autosomal dominant 1
***MECP2***	**Xq28**	**300005**	**312750**	**Rett syndrome**
				**Rett syndrome, atypical**
			**300055**	**Intellectual developmental disorder, X-linked syndromic 13**
			300260	Intellectual developmental disorder, X-linked syndromic, Lubs type
*MEF2C*	5q14.3	600662	613443	Neurodevelopmental disorder with hypotonia, stereotypic hand movements, and impaired language
*NGLY1*	3p24.2	610661	615273	Congenital disorder of deglycosylation 1
*NRXN1*	2p16.3	600565	614325	Pitt-Hopkins-like syndrome 2
*SATB2*	2q33.1	608148	612313	Glass syndrome
*SCN2A*	2q24.3	182390	613721	Developmental and epileptic encephalopathy 11
***SCN8A***	**12q13.13**	**600702**	**614558**	**Developmental and epileptic encephalopathy 13**
			614306	Cognitive impairment with or without cerebellar ataxia
*SLC6A1*	3p25.3	137165	616421	Myoclonic-atonic epilepsy
*SLC9A6*	Xq26.3	300231	300243	Intellectual developmental disorder, X-linked syndromic, Christianson type
***SMC1A***	**Xp11.22**	**300040**	**301044**	**Developmental and epileptic encephalopathy 85, with or without midline brain defects**
***STXBP1***	**9q34.11**	**602926**	**612164**	**Developmental and epileptic encephalopathy 4**
*TBL1XR1*	3q26.32	608628	616944	Intellectual developmental disorder, autosomal dominant 41
*TCF4*	18q21.2	610954	610954	Pitt-Hopkins syndrome
*UBE3A*	15q11.2	601623	105830	Angelman syndrome
*WDR45*	Xp11.23	300526	300894	Neurodegeneration with brain iron accumulation 5
***ZEB2***	**2q22.3**	**605802**	**235730**	**Mowat-Wilson syndrome**

Genes and respective phenotypes marked in **bold** mark those with variants found in this study. Chr, chromosome.

Candidate variants were reviewed by a multidisciplinary group composed of laboratory scientists, bioinformaticians, and clinical geneticists, considering each patient’s clinical information. The SOPHiA DDM™ platform provides variant interpretation based on American College of Medical Genetics/American College of Pathologists (ACMG/AMP) guidelines (Richards et al., 2015). This information was used to select candidate pathogenic, likely pathogenic (P/LP), and variants of unknown significance (VUS) for further analysis. Follow-up investigations included literature searches in PubMed, relevant databases such as RettBase (Krishnaraj et al., 2017) and ClinVar (Landrum et al., 2018), and freely available resources for variant interpretation (https://varsome.com/ and https://franklin.genoox.com). As a result, predicted P/LP variants in genes concordant with the patient’s phenotype were considered informative results. VUS in genes related to the patients’ phenotype are also described. Results were returned and explained to parents and/or guardians in a genetic counselling session by the clinicians in the team.

## Results

### Demographic and clinical characterization

Twenty-eight patients participated in the study, 27 female and 1 male. The median age at inclusion in the study was 11 years, ranging from 18 months to 41 years of age. Demographic and clinical information obtained through the questionnaire and interpreted according to the 2010 RTT criteria (Neul et al., 2010) is summarized in Table 2-Part A. Seven participants (25%) fulfilled clinical criteria for typical RTT, 18 (64%) for atypical RTT, and three (10%) had insufficient information to enable accurate clinical categorization.

**TABLE 2:** Clinical and molecular results.

Part A. Clinical manifestations																Part B. Molecular findings				
ID	Sex	Age	Main Criteria^1^	Supportive Criteria^2^											Exclusion Criteria^3^	Gene	c.DNAVariant	ACMG interpretation^4^	ClinVar ID^5^	OMIM Phenotype
		(years)	I II III IV	1	2	3	4	5	6	7	8	9	10	11	A B	(Transcript)	(Protein Change)			(Number)^6^
**Typical RTT** (n = 7)																				
1	F	9	+ + + +	+	+	-	+	+	+	+	+	+	+	+	- -	*MECP2* (NM_001110792.1)	c.799C>T p.(Arg267*)	P/LP	11829	RTT (312750)
2	F	30	+ + + +	-	+	+	+	+	+	+	+	-	+	+	- -	*MECP2* (NM_001110792.1)	c.799C>T p.(Arg267*)	P/LP	11829	RTT (312750)
3	F	10	+ + + +	-	+	-	+	+	+	-	+	+	+	+	- -	*MECP2* (NM_001110792.1)	c.916C>T p.(Arg306*)	P	11819	RTT (312750)
4	F	7	+ + + +	+	+	-	+	+	+	-	+	+	+	+	- -	*MECP2* (NM_001110792.1)	c.952C>T p.(Arg318Cys)	P	11824	RTT (312750)
5	F	10	+ + + +	+	+	-	+	-	+	+	+	+	+	+	- -	*MECP2* (NM_001110792.1)	c.1174_*7348del p.(Val380_*487del)	P^7^	N/R	RTT (312750)
6	F	27	+ + + +	-	+	-	+	-	-	-	+	+	+	-	- -	*STXBP1* (NM_001032221.3)	c.235C>G p.(Pro79Ala)	VUS	N/R	Developmental and epileptic encephalopathy 4 (312164)?
7	F	3	+ + + +	-	-	-	+	+	+	+	+	+	+	-	- -	N/G	-	-	-	-
**Atypical RTT** (n = 18)																				
8	F	10	+ + + +	+	+	-	+	+	+	+	+	+	+	+	+ +	*MECP2* (NM_001110792.1)	c.239C>G p.(Ser80*)	P/LP	143500	RTT (312750)
9	F	2	+ + - +	+	+	+	+	-	+	-	+	+	+	+	- +	*MECP2* (NM_001110792.1)	c.799C>T p.(Arg267*)	P/LP	11829	RTT (312750)
10	F	9	+ + + +	+	+	-	+	+	+	+	+	+	+	+	+ +	*MECP2* (NM_001110792.1)	c.842del p.(Gly281Alafs*20)	P	95202	RTT (312750)
11	F	12	+ + + +	-	+	-	+	+	-	+	+	+	+	+	- +	*MECP2* (NM_001110792.1)	c.916C>T p.(Arg306*)	P	11819	RTT (312750)
12	F	11	+ + + +	-	+	-	-	+	+	+	+	+	+	+	- +	*MECP2* (NM_001110792.1)	c.952C>T p.(Arg318Cys)	P	11824	RTT (312750)
13	F	11	+ + - +	+	-	-	-	-	-	+	+	+	-	-	- -	*MECP2* (NM_001110792.1)	c.1193_1236del p.(Leu398Glnfs*4)	P	143372	RTT (312750)
14^8^	M	5	+ + + +	-	+	-	+	-	+	-	-	+	+	+	- +	*MECP2* (NM_001110792.1)	c.289C>T p.(Arg97Cys)	VUS	424578	Intellectual developmental disorder, X-linked syndrome (300055)?
15	F	1.5	- - - +	-	-	+	+	+	-	-	-	-	-	+	- -	*GRIN2B* (NM_000834.3)	c.2252T>C p.(Ile751Thr)	P	234500	Developmental and epileptic encephalopathy 27 (616139) or Intellectual developmental disorder, autosomal dominant 6, with or without seizures. (613970)
16	F	21	+ + + +	+	+	-	+	-	+	+	+	+	+	+	+ +	*MADD* (NM_001135943.1)	c.3391A>C p.(Ser1131Arg)	VUS	N/R	Neurodevelopmental disorder with dysmorphic facies, impaired speech and hypotonia (619055)?
																	c.1167_1168del p.(Lys389Asnfs*3)	LP	N/R	
																*TRPM3* (NM_001007471.2)	c.4297dupA p.(Thr1433Asnfs*11)	LP	N/R	Neurodevelopmental disorder with hypotonia, dysmorphic facies, and skeletal anomalies, with or without seizures (620224)
17	F	17	+ + + +	-	-	+	+	+	+	-	+	+	+	+	- N/A	*SMC1A* (NM_00128163.1)	c.3626A>G p.(Asn1209Ser)	Conflicting VUS-B	587912	Cornelia de Lange syndrome type 2 (300590)? or Developmental epileptic encephalopathy 85, with or without midline brain defects (301044)?
18	F	7	+ + - +	-	-	-	+	-	-	-	-	-	+	-	- -	*KCNT1* (NM_020822.2)	c.2213C>T p.(Pro738Leu)	VUS	1011542	Developmental and epileptic encephalopathy 14 (614059)? or Epilepsy nocturnal frontal lobe 5 (615055)?
19	F	25	+ + + +	+	+	-	+	+	+	?	+	+	+	+	- N/A	*SCN8A* (NM_001177984.2)	c.5528A>G p.(Glu1843Gly)	VUS	N/R	Cognitive impairment with or without cerebellar ataxia (614306) or Developmental and epileptic encephalopathy 13 (614558)?
20	F	14	? ? + +	-	+	+	+	+	+	+	+	+	+	+	+ -	N/G	-	-	-	-
21	F	12	+ - + +	+	-	-	+	-	-	+	+	+	+	+	- +	N/G	-	-	-	-
22	F	26	- + + +	-	-	+	+	-	+	-	+	-	-	+	- +	N/G	-	-	-	-
23	F	4	+ + - +	-	+	-	+	-	-	+	+	+	-	-	- -	N/G	-	-	-	-
24	F	41	+ + + +	-	+	-	+	-	+	-	+	+	+	+	- +	N/G	-	-	-	-
25	F	25	+ + + -	-	+	-	+	+	+	+	+	+	+	+	- -	N/G	-	-	-	-
**Participants without phenotypic data (n = 3)**																				
26	F															*ZEB2* (NM_001171653.2)	c.1914C>G p.(Tyr638*)	LP	N/R	Mowat-Wilson syndrome (235730)
27	F															*DDX3X* (NM_001193416.2)	c.341G>T p.(Ser114lle)	VUS	N/R	Intellectual developmental disorder, X-linked syndromic, Snijders-Blok type (300958)?
28	F															N/G	-	-	-	

RTT, diagnostic criteria 2010 (Neul et al., 2010) used to categorize patient presentation: Typical RTT: A period of regression followed by recovery or stabilization with all the four main criteria and none of the exclusion criteria. Supportive criteria are not required, although often present in typical RTT. Atypical RTT: A period of regression followed by recovery or stabilization with two out of the four main criteria and 5 out of 11 supportive criteria.

1 Main Criteria: I. partial or total loss of manual skills, II. partial or complete loss of oral language; III. inability to walk or apraxic gait; IV. Manual stereotypies.

2 Supportive Criteria: 1. Breathing alterations during wakefulness**; 2. Bruxism during wakefulness; 3. Altered sleep pattern; 4. Abnormal muscle tone; 5. Growth retardation; 6. Scoliosis/Kyphosis; 7. Alterations in peripheral vascularization; 8. Small and cold hands and feet; Out-of-context fits of laughter or screaming, 10. Decreased response to pain; 11. Intense eye communication.

3 Exclusion Criteria: A. secondary brain injury, B. Grossly abnormal psychomotor development in first 6 months of life.

N/A. Information not available. N/G no candidate gene, P Pathogenic variant. VUS variant of unknown significance. B benign variant F: Female M: Male.

4 ACMG interpretation per SOPHiA DDM™.

5 ClinVar reviewed October 2023.

6 Source: www.omim.org. “?”unproven diagnosis given VUS interpretation of the identified variant.

7 Although SOPHiA DDM provided a VUS interpretation for this variant, it was considered P given the role of large MECP2 deletions in RTT (Lee et al., 2020).

8 The male patient had severe global developmental delay at the time of participation in the study.

### Molecular results

**FIGURE 1:**
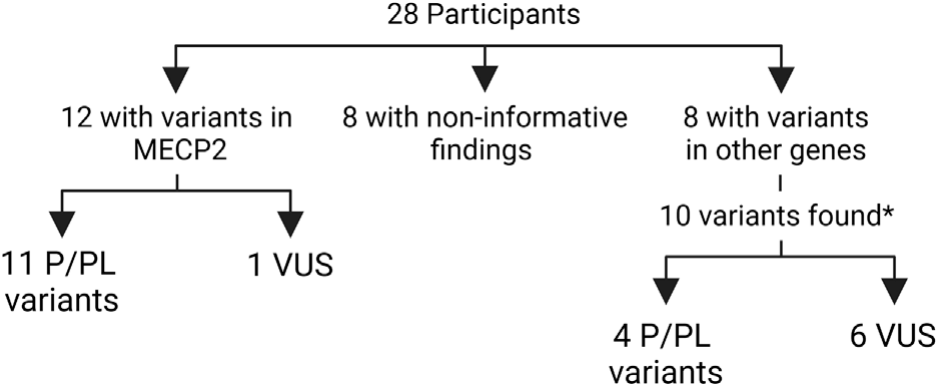
Summary of molecular findings in the participants. *one participant had three variants in two genes P/LP: pathogenic or likely pathogenic VUS: variant of unknown significance.

**FIGURE 2:**
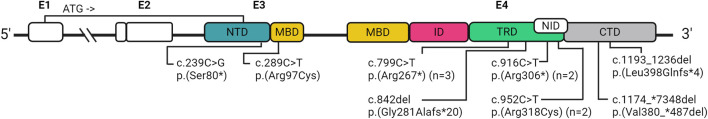
Schematic representation of the location of the identified *MECP2* variants, based on Good et al. (Good et al., 2021) E, exon; NTD, N-terminal; MBD, methyl binding; ID, intervening; TRD, transcription repression; NID, NCoR interaction; CTD, C-terminal domains; n, number of participants with recurrent variants.

In order to identify a causal variant, CES followed by bioinformatics analysis and manual exploration of selected variants was performed. A summary of findings is presented in Figure 1 and individual patient results in Table 2-Part B. Fourteen patients had an informative finding (50%), that is, a P/LP variant in the analyzed protein-coding regions. Eleven patients had P/LP variants in *MECP2* (39.2%), therefore confirming the diagnosis of RTT for them. Three of these *MECP2* variants were recurrent (c.799C>T, c.916C>T, c.952C>T) and seen in two or three patients each, the rest were unique, one of them being a large distal deletion of *MECP2*, the precise breakpoints of which could not be identified with the methodology used (Table 2; Figure 2).

Three patients harbored four predicted PL/P variants in other genes causing neurodevelopmental disorders (*GRIN2B, MADD, TRPM3* and *ZEB2*), leading to alternative diagnoses for them. One of these patients (Table 2, patient 16) had three variants: a LP in *TRMP3*, in addition to a LP variant and a VUS in *MADD*. The phase of the *MADD* variants was not evaluated due to unavailability of parental samples, and further clinical information is needed to assess the possibility of a dual diagnosis.

Seven patients had a VUS, one in *MECP2* and six in other genes related to intellectual disability and other features overlapping RTT: the aforementioned *MADD* in addition to *DDX3X, KCNT1, SCN8A, SMC1A* and *STXBP1*. The male patient had a VUS in *MECP2* inherited from his apparently healthy mother. Parental samples were unavailable to establish the inheritance of the other variants. Seven of the 22 identified P, LP and VUS were not listed in ClinVar at the time of this report.

Finally, eight patients (28.5%) had no evident candidate variant in this study. Of note, no P/LP or VUS were identified in *CDKL5* or *FOXG1*.

Considering the stepwise genomic analysis strategy, six of the nine non-*MECP2* genes were identified with the virtual panel (Table 1, noted in bold lettering), while the other three when assessing variants in the complete CES panel (*KCNT1, MADD* and *TRMP3*), evidencing the benefit of a broad genomic approach.

In order to assess phenotype-genotype correlations, we compared the findings of patients diagnosed clinically as typical and atypical RTT. Five of the seven participants (71.4%) with typical RTT by clinical criteria harbored pathogenic variants in *MECP2,* compared with 6 of 18 (33.3%) of patients with atypical RTT. The recurrent *MECP2* pathogenic variants were seen in patients with both typical and atypical phenotypes. In the atypical RTT group, two patients had an alternative informative diagnosis with P/LP variants in *GRIN2B* and *TRPM3*, and three had VUSs. Of the three patients with incomplete clinical information, one had an informative finding (*ZEB2*), and one had a VUS. The majority (six of eight or 75%) of patients without a molecular finding had been clinically classified as having atypical RTT.

## Discussion

This is the largest genetic study conducted in Chilean patients with suspected RTT syndrome to date and incorporates the use of expanded panel NGS testing, improving over our previous study that performed Sanger sequencing and MLPA for *MECP2* in 14 patients (Aron et al., 2019). We confirmed the diagnosis of RTT in almost 40% of the participants and identified an alternative diagnosis for a subset of others. We expect that this information will be useful for the patients, families and clinicians to guide clinical management.

Our results are similar to other cohorts of patients with RTT or Rett-like phenotypes with a varying proportion of participants receiving a confirmation of RTT, some having alternative findings and others not reaching an etiological diagnosis (Olson et al., 2015; Lopes et al., 2016; Lucariello et al., 2016; Vidal et al., 2017; Vidal et al., 2019a; Vidal et al., 2019b; Cogliati et al., 2019; Iwama et al., 2019; Schönewolf-Greulich et al., 2019). In particular, this has been illustrated in the few studies published from Iberoamerican populations: Lima et al. (Lima et al., 2009) in a study of 105 patients with RTT from Brazil found *MECP2* variants in 60% of them, while Vidal et al. (Vidal et al., 2017) in over 1,500 patients from Spain confirmed the diagnosis of RTT in 30% of them, and found another diagnosis in a similar proportion using different molecular strategies ranging from single gene to exome sequencing.

Although the small sample size limits detailed assessment of phenotype-genotype correlations, *MECP2* variants were identified in higher proportion in patients with typical RTT as in Neul et al. (Neul et al., 2008) and alternative diagnoses or non-informative results were found predominantly in those considered as atypical RTT, similar to the study by Lima et al. (Lima et al., 2009).

The finding of other diagnosis illustrates the complexity of identifying the cause of neurodevelopmental disorders that have overlapping features with RTT, such as developmental delay, developmental arrest, intellectual disability, microcephaly and seizures (Olson et al., 2015; Lopes et al., 2016; Lucariello et al., 2016; Vidal et al., 2017; Vidal et al., 2019a; Vidal et al., 2019b; Cogliati et al., 2019; Iwama et al., 2019; Schönewolf-Greulich et al., 2019) (Table 1). This emphasizes the relevance of detailed clinical evaluations and genomic testing for accurate diagnosis (Manickam et al., 2021), especially for patients with atypical RTT or Rett-like phenotypes. Nevertheless, access to broader genomics tests is limited in low- and middle-income countries, with absent or scarce availability and/or coverage for such testing. Overcoming these challenges is relevant for patients and families, and also for the healthcare systems in Chile and many countries, since testing performed locally is more likely to eventually have financial coverage than testing performed abroad (Encina et al., 2019; Taruscio et al., 2023). Limited mid-throughput NGS equipment is available in several Latin American countries (Torres et al., 2017) and increased during the COVID-19 pandemic for SARSCoV-2 diagnosis and surveillance (Pan American Health Organization, 2022). This situation generates opportunities to further improve diagnostic capabilities for human genetic diseases. Intermediate steps such as gene panels and CES testing can be gradually implemented while capacities for exome and genome sequencing and interpretation are developed at scale.

In addition to improving access for diagnosis, this study identified variants that have not been reported in public databases, showing the value and contribution of including understudied populations in genomic studies, as those in Latin America, to broaden knowledge on genetic and genomic contribution to disease (Sirugo et al., 2019).

Molecular confirmation is particularly relevant for patients with *MECP2* variants. Specific therapies for RTT, such as trofinetide, an analogue of the neuropeptide IGF-1 with anti-inflammatory properties, has successfully completed Phase III clinical trials (Neul et al., 2022) and is now approved by the FDA in the United States for the treatment of RTT (Harris, 2023). Moreover, there are other therapies in various stages of development, such as gene therapy approaches and the application of genome editing using the CRISPR/Cas9 system showing successful *in vitro* repair of mutant *MECP2* in iPSCs and the recovery of its mRNA levels (Croci et al., 2020; Lee et al., 2020).

The limitations of this study include the small sample size, and since participants were recruited through a patient organization after clinical ascertainment, the sample may not represent the broad RTT spectrum in the country. In addition, we relied on the questionnaires and clinical information provided by parents and or guardians, but we did not personally evaluate the patients and families, nor had access to the clinical information that led to the RTT diagnosis, which could lead to over or under-diagnosis. The clinical information questionnaire was designed as a self-guided modality, resulting in some respondents having problems completing it, leaving some questions blank or with unclear answers. The questionnaire was also limited to identifying the presence or absence of RTT criteria and did not capture other elements that could be relevant to suspecting or confirming other potential diagnoses. From the molecular perspective, our study was limited to the search for variants in only 4,000+ genes in the CES panel. Many other genes, not included in the panel, are involved in neurodevelopmental disorders. Furthermore, using this method, we were able to identify intragenic *MECP2* deletions but could not determine the presence of more extensive or complex structural variants involving genes not included in the CES panel, variants in non-coding regions, or mosaicism. Even among the identified variants, this study could not determine the extent of the deletion in patient 5, and whether it corresponds to the recurrent terminal *MECP2* deletion that involves the neighboring *IRAK1* gene, since it is not captured in the panel (Vidal et al., 2019c). Additionally, we were unable to analyze parental samples, because of sample unavailability and funding constraints; this could have enabled to reclassify some of the VUS. Finally, it should be noted that another significant limitation of this study is the scarce reference information available on the genomic variation in Latin American populations, which is a barrier for rare variant analysis and interpretation (Torres et al., 2017).

Working with a patient association was key to developing this study. The motivation originated from their leadership, and the collaborative work not only allowed the research team to reach patients who have this condition more quickly and effectively than an open call to clinicians, but it also helped connect and learn more about biopsychosocial aspects of these conditions, such as patient needs, diagnostic trajectories, and to meet with the families in joint educational activities.

Future steps include implementing trio exome sequencing for these and other families as well as evaluating the clinical impact of reaching a diagnosis. Our study shows that it is feasible to implement such testing in a country with limited genomic resources. The results obtained from collaboration between patients, clinicians and researchers can provide useful input to decision-makers on access and financial coverage of molecular testing for rare disorders, and to subsequently evaluate outcomes resulting from more precise diagnosis.

## Data Availability

The data presented in the study are deposited in the SRA repository, https://www.ncbi.nlm.nih.gov/search/all/?term=PRJNA1011463, bioproject accession number PRJNA1011463.
